# Human Recognition Using Deep Neural Networks and Spatial Patterns of SSVEP Signals

**DOI:** 10.3390/s23052425

**Published:** 2023-02-22

**Authors:** Vangelis P. Oikonomou

**Affiliations:** Information Technologies Institute, Centre for Research and Technology Hellas, Thermi-Thessaloniki, 57001 Thessaloniki, Greece; viknmu@iti.gr

**Keywords:** brain biometrics, human recognition, steady-state visual evoked potential signals, spatial filtering, brain–computer interfaces

## Abstract

Brain biometrics have received increasing attention from the scientific community due to their unique properties compared to traditional biometric methods. Many studies have shown that EEG features are distinct across individuals. In this study, we propose a novel approach by considering spatial patterns of the brain’s responses due to visual stimulation at specific frequencies. More specifically, we propose, for the identification of the individuals, to combine common spatial patterns with specialized deep-learning neural networks. The adoption of common spatial patterns gives us the ability to design personalized spatial filters. In addition, with the help of deep neural networks, the spatial patterns are mapped into new (deep) representations where the discrimination between individuals is performed with a high correct recognition rate. We conducted a comprehensive comparison between the performance of the proposed method and several classical methods on two steady-state visual evoked potential datasets consisting of thirty-five and eleven subjects, respectively. Furthermore, our analysis includes a large number of flickering frequencies in the steady-state visual evoked potential experiment. Experiments on these two steady-state visual evoked potential datasets showed the usefulness of our approach in terms of person identification and usability. The proposed method achieved an averaged correct recognition rate of 99% over a large number of frequencies for the visual stimulus.

## 1. Introduction

Biometrics is the science of establishing the identity of an individual based on the physical and/or behavioral traits of the person in either a fully automated or semi-automated manner [1]. Currently, conventional biometrics, such as fingerprint, face, iris, voice, and DNA, have been extensively studied in the literature and widely adopted in real-life scenarios. However, these biometrics each possess their weaknesses such as being noncancelable, disclosable, and easily stolen, and they do not provide life detection [1,2,3,4]. Brain electrical activity measured at the scalp (through electroencephalography or EEG) can be a potential replacement for the above biometrics since it can be influenced by many genetic factors and presents high individuality among people. Recent studies in the biometric research community support the above statement [5,6]. The EEG has several unique advantages, such as being cancelable, not disclosable, and not easily stolen, and it provides life detection [2].

Our ability to precisely measure physical phenomena related to magnetic resonance and electric currents allows us to observe the brain’s activity related to blood flow in the brain and neurons’ electrical activity. Brain activity can be measured using various imaging techniques, such as functional magnetic resonance imaging (fMRI) [7], functional ultrasound imaging (fUS), magnetoencephalography (MEG), functional near-infrared spectroscopy (fNIRS), and electroencephalography (EEG) [8]. EEG recordings can be acquired using portable and low-cost devices when compared to other brain imaging techniques. EEG is recorded by placing electrodes on the scalp according to the 10–20 international system [8]. The recorded EEG waveforms are classified into different frequency bands (alpha, beta, theta, delta, and gamma waves) ranging (typically) from 0.5 Hz to 40 Hz [8]. While the slowest brain rhythms are related to an inactive brain state, the fastest are related to information processing phenomena [9]. EEG is widely employed for medical purposes, allowing physicians to diagnose epilepsy [10], strokes [11], Alzheimer’s disease (AD) [12], and other brain disorders [13,14]. Additionally, it is used as the basic brain imaging technique for brain–computer interfaces [15,16]. In addition, in recent years, EEG signals have been employed for biometric purposes [2,17].

The adoption of EEG for biometrics purposes has raised some scientific questions related to the overall biometric system. To execute a biometric study using EEG signals, decisions must be made related to (1) the acquisition protocol, (2) features engineering, and (3) decision systems (or classifiers) [2]. The acquisition protocol is strongly related to the EEG device and the EEG responses that we intend to exploit. Two types of EEG devices can be used for biometric purposes: (1) medical-grade systems and (2) low-cost systems [2,18,19,20]. Medical-grade systems contain a large number of electrodes for capturing the EEG, while wet sensors produce high-quality signals. However, the deployment of wet sensors could be impractical in EEG biometric applications. On the other hand, low-cost systems contain a small number of electrodes. In addition, they use dry sensors for reducing the cost and improving the usability of the system [19,20,21].

A crucial decision of an EEG-based biometric system is related to the brain responses used. These brain responses should have properties that are different for each individual. To elicit these brain responses, a particular stimulation protocol must be followed. In the literature, three types of brain stimulation are adopted: resting state, sensory (audio/visual) stimuli, and cognitive tasks. During the resting state, the subject is usually asked to sit on a chair in a quiet environment, either with eyes open or closed. The resting state scheme is the least demanding to generate an external stimulus in terms of needing additional equipment; the users passively produce EEG signals without needing to follow additional instructions during the data collection [4,5]. Most works related to EEG biometrics use EEG signals produced during a resting state [5,21,22,23,24,25,26,27]. However, these systems need to collect a large amount of EEG data. Furthermore, the instruction to users “to rest” may be interpreted in many different ways by the users [4]. On the other hand, a sensory stimuli protocol measures brain responses that are the direct result of a specific sensory event [6,28,29,30,31,32,33,34,35,36]. A drawback of this approach is the need for external devices to produce the stimulus for the generation of the required EEG signals, resulting in a complicated system. Finally, cognitive stimuli could be used as an alternative to the above protocols. A cognitive stimulus involves directing the subject(s) to perform various intentional tasks during the data collection [20,24,37,38,39]. However, cognitive task stimuli also suffer from the ambiguity problem similar to the resting state case [4].

Feature engineering from EEG signals plays a significant role in the person identification problem. Hence, EEG features have been extracted from many different and divergent domains, such as the time domain or brain connectivity (i.e., graphs) domain. Extraction methods based on auto-regressive (AR) modeling, power spectral density (PSD) energy of EEG channels, wavelet packet decomposition (WPD), and phase locking values (PLV) have been used extensively in person identification with EEG signals. In the time domain, the most used features are based on AR modeling. In particular, AR coefficients, either on each EEG band or over the entire EEG frequencies band, have been extracted from resting state EEG signals [5,21,24] attention tasks [24], steady-state visual evoked potential (SSVEP) [35], and visual stimulation [40]. Additionally, features based on energy or statistical measures of a time series were used in [27,28,33,34]. In addition, raw EEG time series, after some basic filtering, were used as input (hence features) in deep learning (DL) schemes [28,36]. Furthermore, to exploit characteristics and structures over EEG’s frequencies, PSD features were extracted for various tasks [20,22,25,26,30] related to EEG biometrics. Since, the time domain and frequency domain provide valuable information about EEG signals, it is natural to exploit the time–frequency distribution using wavelet-based features [24,29]. Finally, in recent years, the connectivity between brain areas has been studied extensively in EEG biometrics. For example, in [24,26,37] PLV values were used to model the brain’s connectivity, while in [23,25,31] functional connectivity (FC) features, such as eigenvector centrality, were proposed for person identification using EEG signals. From the above analysis, we can observe a limited use of spatial patterns for EEG-based person identification.

After the features engineering step, the next step of an EEG biometric system involves the classifier. In the EEG biometric literature, various classifiers have been used, which can be divided into four large families: similarity-based (or distance-based) methods, kernel-based methods, discriminant analysis methods, and neural networks [2,4]. One representative example of the similarity-based method is the k-nearest neighborhood (kNN) classifier. The k-NN classifier compares the similarity (or the distance) between the template feature (or training) samples and the query (or test) samples. The kNN has been used widely in EEG biometrics. In [29], short-time Fourier transform (STFT)-based features were extracted from SSVEP signals and used as input into a kNN classifier, while in [30] PSD features were utilized. In addition, in [34], statistical features (i.e., normalized variances) extracted from SSVEP signals were fed to a kNN classifier. Finally, distance-based classifiers have been used to identify a person [5,21,23,26].

Kernel-based methods are based on the “kernel” trick, where the original finite-dimensional space is mapped into a space with a much larger dimension. Here, the assumption is that the distinction between persons’ identities in this high-dimensional space is easier to determine. The support vector machine (SVM) is the most representative kernel-based method. The SVM classifier was adopted in many EEG biometric studies to distinguish between persons [20,21,27]. In contrast to the SVM and the “kernel” trick, linear discriminant analysis (LDA) uses hyperplanes to separate the data of different classes by projecting the data into a lower dimensional space. Furthermore, LDA assumes that classes are distributed normally with equal class covariance. LDA is one of the most popular classification algorithms in EEG biometrics [19,21,33,37,40].

In the field of EEG biometrics, neural networks (NN), and especially deep NN, have also received attention. An important difference between the various DNN approaches is related to the EEG features used as input to the NN: raw EEG time series or extracted EEG features. Convolutional NNs (CNNs) were used in [36], where the input to the CNN was raw EEG time series, while [24] used CNN with raw EEG as input as well as CNN with various input features, such as AR coefficients, wavelets, and PLV values. Furthermore, adversarial CNNs were used in [28] with raw EEG time series as input. In [41], CNNs with PSD features as input were used to discriminate between persons. Overall, Deep NNs are valuable to identify a person using EEG signals. However, the quantity of data and time required for training efficient deep NNs is a major concern for their effective widespread deployment in EEG biometric systems.

SSVEP signals have been rarely used for the identification of a person. However, SSVEP-based person identification systems should leverage the advantages of current SSVEP protocols applied in BCI systems, such as the ability to design a speller. In [29], the authors proposed an SSVEP-based biometric system, with one stimulus frequency that varied from 6 to 9 Hz. The authors collected SSVEP signals from five users. To decide about the user identity, features based on short time Fourier transform and the standard EEG bands were extracted. Then, these features were used as input to a kNN classifier. In [34], eight subjects participated in an experiment, and six stimulus frequencies were used for the production/elicitation of SSVEP signals. Normalized variances were obtained for each stimulus frequency and each channel and were used as features. Furthermore, the kNN classifier was used to decide the identity of a user. In [35], SSVEP signals from twenty-five subjects were used. Additionally, four different stimulus frequencies were used to evoke the SSVEP responses. Mel-frequency cepstral coefficients (MFCCs) and autoregressive (AR) reflection coefficients were used as discriminative features. The Manhattan (L1) distance was used to evaluate the similarity between users, and the decision was made to use the minimum among the distances. In [36], two SSVEP datasets were used (5 and 12 individuals) with 3 stimulus frequencies. To decide about the identity of a user, the authors used convolutional neural networks on raw SSVEP signals and enhanced SSVEP signals, where the enhanced signals were obtained by using canonical correlation analysis (CCA).

We can observe that spatial patterns of individuals from SSVEP signals have not been utilized, and all reported works applied to a limited number of individuals and stimulus frequencies. The number of stimulus frequencies is an important aspect of the overall system since it defines and restricts our design’s options. A small number of stimulus frequencies does not give us the ability to design a biometric system using properties from an SSVEP speller. Using an SSVEP speller as a basic component of a biometric system, we can design a biometric system that simultaneously recognizes the user through SSVEP responses and passwords. Concerning EEG biometrics, and not only SSVEP-based biometrics, we can observe the limited use of spatial filtering approaches. Spatial patterns of EEG have not been investigated extensively (at least to the author’s knowledge), except for [33] who used spatial filters in auditory evoked potentials (AEP). The novelties and contributions of our work are related to:

- The use of SSVEP signals for person identification;
- A new DL-based framework for person identification (using SSVEP signals);
- A new kind of spatial features for EEG person identification using the filterbank common spatial patterns (FBCSP) methodology in multi-class problems;
- An extension of a current SSVEP-based biometric system to a larger number of stimulus frequencies. This number is sufficient to design a speller.

This paper is organized as follows. In Section 2, we provide information about the SSVEP datasets and the proposed methodology for person identification. More specifically, detailed descriptions of the spatial filtering method and the architecture of neural networks are provided. After that, in Section 3, we provide information about the experimental settings of our experiments. Then, in Section 4, we present a comprehensive comparison of our approach with well-known classifiers. Finally, in Section 5, we provide a discussion and concluding remarks related to our work and its future directions.

## 2. Materials and Methods

### 2.1. SSVEP Datasets

In our work we have used two benchmarks SSVEP datasets for PI, the *Speller* dataset (http://bci.med.tsinghua.edu.cn/download.html, accessed on 9 June 2017) and the *EPOC* dataset (https://physionet.org/content/mssvepdb/1.0.0/, accessed on 3 September 2016). Next, we provide a short description of each dataset. The *Speller* dataset [42] provided us with SSVEP responses from 35 subjects, and the number of different stimuli was 40. More specifically, the stimulation frequencies range from 8 Hz to 15.8 Hz with an interval of 0.2 Hz, and the phase difference between two adjacent frequencies was 0.5π. The EEG signals were acquired using a Synamps2 EEG system (Neuroscan, Inc., Charlotte, NC, USA) device with 64 channels based on an extended 10–20 system. In our study, we utilized the nine channels covering the occipital and parietal–occipital areas ($P_{z}$, $PO_{5}$, $PO_{3}$, $PO_{z}$, $PO_{4}$, $PO_{6}$, $O_{1}$, $O_{z}$, $O_{2}$). Each subject completed 6 blocks, where in each block, the subject was looking at the visual stimuli for 5 s. Furthermore, in each block, the subject was looking at 40 different visual stimuli, one for each target. After the extraction of EEG trials, the signals were band-pass filtered from 7 to 90 Hz with an infinite impulse response (IIR) filter using the *filtfilt* function from MATLAB. In the *EPOC* dataset [43], EEG signals were acquired during an SSVEP-based experimental protocol, using the Emotiv EPOC device. This particular device has 14 channels and a sampling rate of 128 Hz. Furthermore, the stimulation frequencies were 6.66 Hz, 7.50 Hz, 8.57 Hz, 10.00 Hz, and 12.00 Hz. Each subject completed 20 trials for each of the 5 targets. We filtered EEG signals using a band-pass filter from 5 Hz to 45 Hz. More information about this dataset can be found in [43]. The selection of these particular datasets will give us the ability to study PI using SSVEP signals when we have a large number of participants, a large number of stimulation frequencies and frequencies’ range, and SSVEP signals from wearable and portable EEG devices.

An SSVEP dataset is a collection of multichannel EEG trials ${\left\{X_{1}^{\left(s\right)},X_{2}^{\left(s\right)},\cdots ,X_{M}^{\left(s\right)}\right\}}_{s=1}^{N_{s}}$, where *M* is the number of trials of a participant, (s) is the index of the participant, and $N_{s}$ is the number of participants (or classes). Each $X_{m}^{\left(s\right)},m=1,\cdots ,M,s=1,\cdots ,N_{s}$ is a matrix of $N_{ch}\times N_{t}$, where $N_{ch}$ is the number of channels and $N_{t}$ the number of samples. Additionally, we assume that the multi-channel EEG signals are centralized since, in practice, the EEG trials are band-pass filtered or detrended. Furthermore, for purposes of PI, we assume that all SSVEP responses are acquired using visual stimuli that are flashing at the same frequency.

### 2.2. Spatial Filtering and Filterbank Common Spatial Patterns

Given the EEG trial X, a spatial filter $w:N_{ch}\times 1$ is the weights describing the linear combination of EEG channels with the overarching goal to produce a spatially filtered EEG trial (i.e., $X^{T}w$) with higher SNR than the original EEG trial (i.e., X). Typically, we can determine spatial filters manually or empirically. Typical spatial filters of this kind are the bipolar combination, the common average reference spatial filter, and the Laplacian spatial filter. However, these kinds of filters do not make efficient use of any prior information about the problem or any subject-specific information. Hence, many works have studied how to learn efficient spatial filters [16,44,45]. Typically, these spatial filters use labeled data and provide much better performance.

One of the most used spatial filtering methodologies is the filterbank common spatial patterns (FBCSP). In the FBCSP method, the EEG trial X is filtered by a filterbank of *B* filters, producing *B* filtered trials, $X^{\left(b\right)},b=1,\cdots ,B$. FBCSP aims to learn the spatial filters in *B* frequency bands (b=1,⋯,B) by maximizing the variance of band-pass filtered EEG signals from one condition (or class) while minimizing their variance from the other condition (or class) [46,47]. Note here that the FBCSP method requires knowing if a trial belongs to the first or the second condition; hence, it is a supervised learning method. The spatial filters are obtained by maximizing (or extremizing) the following function [44,45,46,47]:

(1) $$\begin{array}{c} J\left(w_{b}\right)=\frac{w^{T}C_{1}^{\left(b\right)}w_{b}}{w_{b}^{T}C_{2}^{\left(b\right)}w_{b}},b=1,\cdots ,B \end{array}$$

where *T* denotes transpose, and $C_{i}^{\left(b\right)}$ is the covariance matrix of the *i*-th condition in the *b*-th frequency band. The above maximization problems are equal to maximize $w_{b}^{T}C_{1}^{\left(b\right)}w_{b}$ subject to the constraints $w_{b}^{T}C_{2}^{\left(b\right)}w_{b}=1$, which, finally, are equivalent to the generalized eigenvalue problems $C_{1}^{\left(b\right)}w_{b}=\lambda C_{2}^{\left(b\right)}w_{b},b=1,\cdots ,B$. So, the spatial filters $w_{b}$ are the generalized eigenvectors of the above problems corresponding to the largest eigenvalue. However, in classification settings, it is preferable to use eigenvectors from both ends of the eigenvalue spectrum as spatial filters [44,46]; hence, in the end, for each frequency band we have a matrix $W_{b}:N_{ch}\times 2p$ of spatial filters, where 2p is the number of spatial filters. A common practice to determine the number of spatial filters is to choose the 2p eigenvectors corresponding to the *p* largest eigenvalues and the *p* smallest eigenvalues [46].

After finding the spatial filters, we used them to spatially filter the trial corresponding to the *b*-th band, $X_{i}^{\left(b\right)}$. Hence, we obtained the spatially filtered trial, $Z_{i}^{\left(b\right)}=X_{i}^{\left(b\right)}W_{b}$, from which we calculated the variances in each row (i.e., in each “new” channel). Hence, a CSP-related feature vector for the *b*-th band $f_{i}^{\left(b\right)}=\left[f_{1}^{\left(b\right)},f_{2}^{\left(b\right)},\cdots ,f_{2p}^{\left(b\right)}\right]$ is constructed as: $f_{j}^{\left(b\right)}=\log\left(\mathrm{var}\left(Z_{i}^{\left(b\right)}\left(j,:\right)\right)\right),j=1,\cdots ,2p$. Furthermore, by collecting all feature vectors, $f_{i}^{\left(b\right)},b=1,\cdots ,B$, in one vector, we have $f_{i}^{\left(b\right)}=\left[f_{i}^{\left(1\right)},\cdots ,f_{i}^{\left(b\right)},\cdots ,f_{i}^{\left(B\right)}\right]$, with size (B·2·p)×1. The above analysis was performed by assuming the discrimination between two conditions. However, in our problem, the conditions are equal to the number of persons ($N_{p}$) that we want to identify. In this case, we adopt the one-versus-rest approach [47] which computes the CSP features that distinguish each person from the rest of the persons. More specifically, we compute spatial filters for each person against all others persons. Then, we project the EEG signals on all these spatial filters and calculate the variances as in the binary case described above. In the multiclass extension, the size of the features’ vector is $(N_{p}\cdot B\cdot 2\cdot p)\times 1$.

### 2.3. Deep Feedforward Neural Network (DFN)

The multilayer perceptron (MLP) is a fundamental example of a deep neural network. The architecture of an MLP consists of multiple hidden layers to capture more complex relationships that exist in the training dataset. Another name for the MLP is the deep feedforward neural network (DFN) [48,49]. In DFN, we stack layers of neurons together to create a hierarchy of deep representations. By doing this, the network learns what features are relevant and also learns the weights of the network that best approximate the target function. Initially, the hidden layers closer to the input layer learn a simple set of features, which then grow to increasingly complex features as information flows to deeper layers of the network, to capture the mapping between the inputs and the target. The major task in deep learning is to learn representations of input data to improve the classification. More specifically, the input data are mapped into new (deep) representations by applying non-linear transformations to them. Composing multiple non-linear transformations result in hierarchical representations of input with an increased level of abstraction due to transformations. Given sufficient data, deep NNs can learn high-level feature representations of inputs through the composition of multiple hidden layers [50].

**Figure 1:**
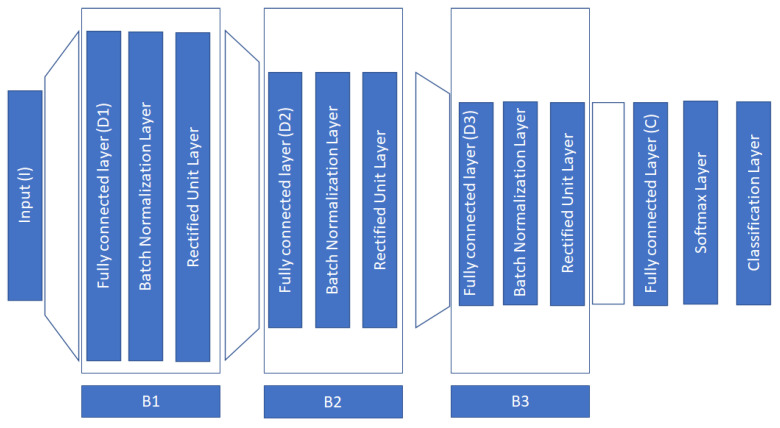
Architecture of the proposed DFN.

Our network consists of three blocks, where each block consists of a fully connected layer, a batch normalization layer, and a rectified unit layer [48]. The purpose of the fully connected layer is to provide transformations of the layer’s inputs which are then fed into two non-linear operators, the batch normalization operator, and the rectified unit operator. The goal of batch normalization is to ensure zero mean and unit variance for the activations across the samples in a minibatch. This operator positively affects various aspects of the network, such as training speed, stability, and sensitivity to the learning rate, and additionally, it acts as a regularizer [48]. During our experiments, we verify its usefulness for our analysis/purpose. In Figure 1, we depict the architecture of the proposed network.

The input to the network is the feature vectors $f_{i}\in {ℜ}^{I}\left(I=N_{p}\cdot B\cdot 2\cdot p\right)$. In these features vectors, we apply a number of non-linear transformations described by:

(2) $$\begin{array}{ccc} z_{j} & = & W_{j}o_{j-1}+b_{j},W_{j}\in {ℜ}^{D_{j}\times D_{j-1}},b_{j}\in {ℜ}^{D_{j}} \end{array}$$

(3) $$\begin{array}{ccc} o_{i} & = & ReLU\left(BatchNorm\left(z_{j}\right)\right)\in {ℜ}^{D_{j}}, \end{array}$$

where j=1,2,3, $D_{0}=I$, $o_{0}=f_{i}$, and ReLU(·) and BatchNorm(·) represent the non-linear operations of rectified linear unit and batch normalization. At the classification block, we apply the softmax function, and for adjusting the weights $W_{j}$, we use the Adam optimizer to minimize the cross-entropy loss function [36,48] between the predicted labels, ${\hat{y}}_{c}$, and the true labels, *y*: $f\left(y,\hat{y}\right)=-\sum_{c=1}^{C}y_{c}\log{\hat{y}}_{c}$.

In our approach, we further constrain the dimensionality in each layer according to: $D_{0}=I,D_{1}>>D_{0},$$D_{2}<D_{1}$, $D_{3}<D_{2},D_{3}>C,D_{4}=C$. The above restrictions in dimensions follow the current logic: first, we project the input data into a much larger dimension inspired by the kernel trick, where we hope that this guided representation into much higher dimensions will provide us with more discriminative power. Then, in the subsequent layers, we slowly decrease the dimension in each layer to encode the representation of the first layer. Ablation studies in the experimental section experimentally verify our intuition. In Table 1, we provide the configuration details related to the implemented architecture and the network’s hyperparameters.

**Figure 2:**
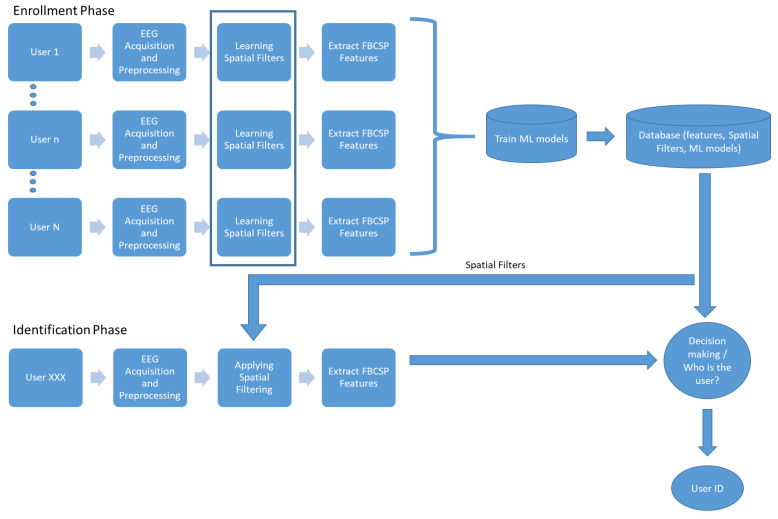
Architecture of the proposed person identification system.

Closing this section, we provide the overall proposed architecture of the system for person identification in Figure 2. The proposed system consists of four main components: the acquisition and preprocessing of EEG signals, the learning of spatial filters, the extraction of FBCSP features, and the training of the DFN model. In the enrollment phase, multi-channel EEG signals are collected from each user under the SSVEP protocol and fed into the spatial filtering module, which constructs the spatial filters and extracts the FBCSP features. Then, the DFN model is trained. In the database, we store the DFN model, the spatial filters, and the FBCSP features. In the authentication phase, we collect the EEG signals, apply the spatial filters to extract the FBCSP features, and finally, we use the DFN model to determine the identity of the user.

## 3. Experiments

### 3.1. Performance Measures

Since the person identification task is a one-against-all classification task, we choose as the performance measure the correct recognition rate (CRR). The CRR measure is a well-known performance measure that is extensively used for person identification purposes, and it is defined as the average over the diagonal elements of the resulting confusion matrix [26]. However, besides the conventional measure of CRR, other factors affecting the overall usability of the system should be taken into consideration for assessing the practical usability of any reported EEG-based recognition systems. In this direction, the usability measure was proposed in [4] which is defined as:

(4) $$U=\frac{N\times CRR}{Tr+K\times Te}$$

where *N* is the number of subjects, *K* the number of electrodes, Tr the duration of the training set, and Te the duration of the test set.

### 3.2. Experimental Settings

Typically, in an SSVEP experiment, a number of experimental settings must be defined and reported. More specifically, it is important to report the following settings:

- $N_{p}$ number of subjects;
- $N_{B}$ number of blocks;
- $N_{f}$ number of stimuli frequency;
- $N_{ch}$ number of EEG channels;
- $D_{tr}$ trial duration;

which for the SSVEP dataset are provided in Table 2. Furthermore, to train the reported classification schemes, we adopt the leave-one-block-out (LOBO) cross-validation approach, where the block contains an SSVEP trial from each subject (total $N_{p}$ trials). We compare our method with:

- SVM-FBCSP: the SVM classifier with a linear kernel where FBCSP features are used for performing the person identification;
- kNN-FBCSP: the kNN classifier with FBCSP features.

**Figure 3:**
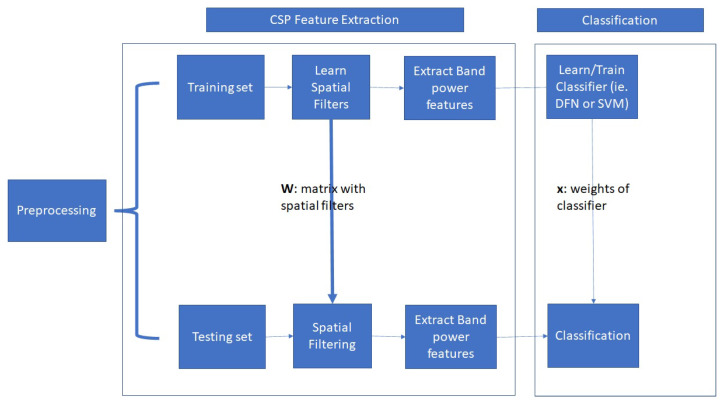
Data analysis procedure.

Furthermore, in Figure 3 we schematically depict the overall data analysis procedure. First, the EEG data are temporally filtered. Then, the spatial filters are learned to extract the FBCSP features, and finally the FBCSP features are used as input to the classifier. For the Speller dataset, the SSVEP signals are band-pass filtered from 8–30 Hz, while in the case of the EPOC dataset, the frequency range of the filter was from 4–30 Hz. Additionally, for the Speller dataset we use the 9 channels from occipital and parietal-occipital areas ($P_{z}$, $PO_{5}$, $PO_{3}$, $PO_{z}$, $PO_{4}$, $PO_{6}$, $O_{1}$, $O_{z}$, $O_{2}$), while for the EPOC dataset, we use all available 14 channels, covering the entire brain.

## 4. Results

SSVEP signals are generated by stationary localized sources and distributed sources in the parietal and occipital areas of the brain. Our overarching goal in these experiments is to examine if SSVEP signals contain information that can be used to discriminate individuals. Furthermore, we examine if this information contains distinguishable spatial patterns between individuals. In Table 3, we provide the results of our first experiment. Our intention in this experiment was to examine if our algorithm could identify persons using SSVEP signals from various frequencies of the stimulus. In Table 3, we provide the performance of all reported methods. In this experiment, the length of an SSVEP trial is 5 s. The DFN method provides us a better person recognition rate than all other methods. Importantly, this observation could be extended to a wide range of frequencies. The DFN method presents better performance for 40 different stimuli frequencies ranging from 8 to 15 Hz. This is reflected in the average CRR over all stimuli. We can see that, on average, the DFN has a CRR of 99% compared to 94% and 93% of SVM-FBCSP and kNN-FBCSP, respectively. Additionally, it is important to note that for some stimuli frequencies, the CRR is perfect (100%), see for example the CRR for 9 Hz and 15 Hz. The above observations indicate that the DFN method in conjunction with carefully chosen stimuli frequency could provide excellent person identification. To examine if the observed differences in Table 3 are statistically significant, we conducted one-way ANOVA to compare the effect of classification methods on CRR values. We performed comparisons between all reported methods. There was a significant difference in accuracy among the classification methods at the *p* < 0.05 level for the three methods F(2,117) = 279, *p* < 0.001. Furthermore, post hoc analysis revealed that the proposed method had significantly different accuracy from the other methods. These results indicate that the chosen method for PI has a statistically significant effect on the final model’s performance. The above experimental results were implemented using Matlab on a PC with Intel(R) Core(TM) i5-4690K CPU @ 3.50 GHz, 16 GB RAM, and 64-bit Windows 10 OS. In a typical case, the spatial filter training took up to 1.38 s, while the network training took up to 11.29 s.

In general, it is desirable to use SSVEP signals of small duration because they contribute to the design of EEG-based biometric systems that are more comfortable with a better user experience. Hence, we performed additional experiments to see the behavior of methods when we have different durations of SSVEP signals. In Table 4, we provide the averaged CRR over all stimuli frequencies for various durations of SSVEP trials. We can observe that the DFN method provides the best recognition rate among all methods for all trials’ durations. Additionally, we conducted one-way ANOVA to compare the effect of the method on CRR values. There was a significant difference in accuracy among the classification methods at the *p* < 0.05 level for the 3 methods F(2,27) = 5.89, *p* = 0.008. Furthermore, post hoc analysis revealed that between the DFN and other methods, we had significantly different accuracy; however, we did not find significantly different accuracy between the SVM-FBCSP and the kNN-FBCSP. The above results indicate that the DFN presents better performance than the SVM-FBCSP and the kNN-FBCSP even if we choose a different duration of a trial in our experimental settings.

In our study, we also evaluated the usability of the comparative methods to have a more compact view of the proposed algorithms, as well as to provide comparisons with other EEG-based PI systems reported in the literature. In Table 5, we provide the obtained results when we calculate the averaged *U* measure for various trial durations. The best *U*-value (101.2288) is obtained from the DFN method, where the duration of the trial is 0.5 s. Comparing our approach with those reported in [4] concerning the *U*-measure, we can see that our approach presents the best performance. It is important to note here that the significant aspect that gives us the ability to have better performance than other methods reported in [4] is that our method presents high CRR for a very small duration of EEG trials. Clearly, other methods have presented very high CRR, similar to ours; however, these methods need larger EEG trials and more channels.

We conducted similar experiments using a second SSVEP dataset (the *EPOC* dataset) to see the behavior of our approach in different settings. It is important to note here that for the acquisition of SSVEP signals, the portable Epoc device was used. In Table 6, we provide the obtained CRR values for various frequency stimuli. We can observe that the DFN method presents the best performance among competitive methods. Furthermore, in Table 7, we provide the averaged CRR values for various durations of EEG trials. Again, the DFN method presents the best performance for all trials’ duration except that of 1 s. Finally, in Table 8, we provide the utility values. We can see that the DFN method provides us with the best *U* value when the duration of the trial is 0.5 s. Comparing the provided results on the two SSVEP datasets, we can see that all methods present the best performance (using either the CRR measure or the *U* measure) in the case of the Speller dataset. This is something that we expect to some degree since, due to experimental settings and the used EEG devices, the EEG signals in the Epoc dataset are very noisy. However, we must not underestimate the results in the *EPOC* dataset since they provide indications that a portable SSVEP-based PI can be designed and used for real applications outside the lab.

It is typical when a new DL architecture is proposed to conduct an ablation study to see how the various building layers affect the overall performance of the proposed neural network. In our study, we conducted one such study by removing major blocks of our architecture as those described in Figure 1. More specifically our approach contains three major blocks (B1, B2, B3). We conducted experiments where we removed each block starting from the “top” of the deep architecture. The obtained results are provided in Table 9. From these results, we can observe the significance of block B1. This block is responsible to project the input to a much higher dimension. The inclusion of the B1 block in the deep architecture gives a significant boost to the performance of the final neural network.

## 5. Discussion and Conclusions

In this study, a large number of subjects were used for the design of an EEG-based biometric system. Furthermore, a large number of SSVEP stimuli were used to test the performance of the proposed approach. The results provide evidence that a wide range of frequencies can be used for the stimulus without sacrificing the performance of the system. In addition, the experiments with the EPOC device provide evidence that wearable EEG devices could be used to detect the SSVEP responses for biometric purposes. Finally, an additional characteristic of our approach is the CSP features that are used for the first time to identify persons. The CSP algorithm gives us the ability to design personalized spatial filters to identify a person. In addition, we can observe that the spatial patterns of the brain can have discriminative properties when we intend to identify persons.

This work demonstrates that the proprietary use of DFN outperformed the more modern neural network architectures such as CNN. For person identification, the proposed DFN gained the consistently highest performance among the evaluated methods. Additionally, it presented better performance than CNN architectures in SSVEP-related biometric scenarios [36]. It is evident that the proper choice of features and the deep architecture of MLP can leverage the performance of conventional neural networks. Furthermore, modern deep learning architectures are ordinarily performed on high-performance computing facilities due to the the large size of the input features and the complexity of its model. Our work has shown that traditional MLP with deep layers and small input size can provide efficient performance and tackle the computation requirement limitation of modern approaches.

In the BCI community, a BCI speller is a tool for measuring and converting brain signals to commands without the participation of the peripheral nervous system. Additionally, when an SSVEP-based BCI speller is adopted, we can design a graphical interface that resembles the traditional QWERTY keyboard. This feature of the SSVEP speller gives us the ability to design a two-step authentication system. In one such system, the user inserts the PIN through the SSVEP speller, while the authentication system simultaneously recognizes the user through his/her SSVEP responses. This system combines two modalities for the authentication process: what you know and who you are [33]. Furthermore, such systems could be very helpful in situations where the user is not able to introduce the password by using conventional means such as keyboards. These situations rise when people deal with situations that affect the central nervous system, such as Parkinson’s disease, amyotrophic lateral sclerosis (ALS), and tetraplegia. However, in the design of SSVEP-based biometric systems, we must consider the fatigue which is evoked by the flicker [51]. To alleviate this characteristic, it is important to design systems where the duration of SSVEP signals is small. In addition, problems are raised due to the SSVEP illiteracy phenomenon where some people do not produce valuable SSVEP responses [52]. Finally, SSVEPs can elicit epileptic responses to luminance or chromatic stimuli, and special care must provided with respect to this issue [51].

The non-stationarity of EEG signals is considered a major challenge in EEG signal processing, and a lot of effort has been devoted to accommodating this issue [53]. EEG non-stationarities can be raised due to neurophysiological mechanisms related to learning, plasticity, and aging mechanisms and due to experimental conditions related to the recording quality, device configurations, and small changes in electrode locations. Hence, a significant issue that affects the performance and the acceptability of an EEG-based biometric system is the permanence of EEG features across time. To attack this effect, EEG signals must be collected across time for the particular acquisition protocol. In the literature, many efforts have been performed under this assumption; however, the time interval between sessions is one month, making it difficult to study the performance of EEG-based biometric systems in larger time intervals (larger than a year). A solution to the above problem could be the re-calibration of the EEG database in specific time intervals. Furthermore, from an algorithmic perspective, algorithms based on lifelong learning could be very useful in EEG-based biometric systems. This type of algorithm can take into consideration the time-varying distribution of EEG features and the covariance shifts of features [53]. Future directions of our study could include extensions of our approach to fit the authentication and continuous authentication scenarios [24]. In addition, the adoption of multi-modal systems utilizing portable EEG devices and Gait analysis using IoT devices [54] could provide valuable information related to the identification of a person.

In concluding this paper, it is important to emphasize that we have proposed a new methodology for person identification using SSVEP signals. The new methodology is based on the combination of personalized spatial filters and deep neural networks. The provided results in two SSVEP datasets show the usefulness of our approach, especially if we take into account the usability measure. This measure provides us with a way to compare our methodology with other brain biometrics methodologies. Comparisons of our approach with other classification schemes, such as SVM-FSCSP and kNN-FBCSP, show the superiority of our approach. Additionally, the results from the ablation study justify the proposed deep architecture over other deep architectures. Finally, comparisons of our approach with other methodologies in terms of *U* measure show the basic advantage of our approach which is the high recognition rate using EEG signals of very small duration (0.5 s).

## Figures and Tables

**Table 1:** Details of the network’s configuration.

Parameter	Value
*I*	Dimensionality of the FBCSP feature vector
$D_{1}$	500
$D_{2}$	100
$D_{3}$	50
$D_{4}$	*C* (number of persons)
Learning Rate	0.001
Batch Size	32
Epochs	200
OutputNetwork	Choose network with the lowest validation loss

**Table 2:** Experimental settings for the two SSVEP dataset.

Parameter	Speller	EPOC
$N_{p}$	35	11
$N_{B}$	6	20
$N_{f}$	40	5
$N_{ch}$	9	14
$D_{tr}$	5 s	5 s

**Table 3:** Results using the Speller dataset. CRR (%) is provided for each frequency at TW = 5 s. Note: The bold value indicates the best value on each frequency.

Frequency Stimulus (Hz)	DFN	SVM-FBCSP	kNN-FBCSP
8	**99.5238**	92.8571	92.3810
9	**100.0000**	94.7619	94.2857
10	**98.0952**	96.6667	94.7619
11	**98.5714**	94.7619	93.3333
12	**98.5714**	94.7619	94.7619
13	**99.0476**	95.2381	95.7143
14	**99.0476**	94.2857	91.4286
15	**100.0000**	95.7143	94.2857
8.2	**99.0476**	95.2381	93.8095
9.2	**99.0476**	95.2381	93.3333
10.2	**99.5238**	97.1429	96.1905
11.2	**99.5238**	96.6667	93.3333
12.2	**98.5714**	94.2857	93.8095
13.2	**99.0476**	95.7143	91.4286
14.2	**99.0476**	96.1905	92.3810
15.2	**99.5238**	95.7143	90.4762
8.4	**100.0000**	94.7619	93.3333
9.4	**99.5238**	93.3333	94.2857
10.4	**98.5714**	95.2381	93.3333
11.4	**99.5238**	94.7619	95.2381
12.4	**100.0000**	94.7619	94.2857
13.4	**98.0952**	94.2857	93.8095
14.4	**100.0000**	95.7143	95.7143
15.4	**99.5238**	91.9048	90.9524
8.6	**99.0476**	96.1905	92.3810
9.6	**98.5714**	95.7143	90.4762
10.6	**98.5714**	96.6667	92.3810
11.6	**99.5238**	93.8095	92.8571
12.6	**99.0476**	94.2857	92.8571
13.6	**97.6190**	95.7143	92.3810
14.6	**98.0952**	96.6667	89.5238
15.6	**97.1429**	93.8095	90.9524
8.8	**100.0000**	95.7143	93.8095
9.8	**99.0476**	94.7619	93.8095
10.8	**99.0476**	91.4286	91.9048
11.8	**99.5238**	94.2857	95.2381
12.8	**99.0476**	94.2857	92.8571
13.8	**98.0952**	91.9048	93.3333
14.8	**98.5714**	95.2381	94.7619
15.8	**98.5714**	93.8095	88.0952
average	**99.0238**	94.8571	93.1071

**Table 4:** Results using the Speller dataset. The provided CRR (%) values are averaged over all frequencies for various TW. Note: The bold value indicates the best value on each TW.

TW (s)	DFN	SVM-FBCSP	kNN-FBCSP
0.5	**85.3214**	74.7024	74.7262
1.0	**95.1429**	85.6905	84.9524
1.5	**97.0238**	88.5595	87.7738
2.0	**98.2024**	91.3452	89.9286
2.5	**98.5238**	92.4762	90.8452
3.0	**98.8333**	93.3571	92.0119
3.5	**98.8810**	94.1429	92.3571
4.0	**99.0714**	94.5833	92.6786
4.5	**99.0357**	94.7024	92.8095
5.0	**99.0238**	94.8571	93.1071

**Table 5:** Results using the Speller dataset. The utility (U) values are averaged over all frequencies for various TW. Note: The bold value indicates the best value among all TWs.

TW (s)	DFN	SVM-FBCSP	kNN-FBCSP
0.5	**101.2288**	88.6300	88.6582
1.0	97.9412	88.2108	87.4510
1.5	88.2035	80.5086	79.7944
2.0	79.9322	74.3507	73.1977
2.5	72.5965	68.1404	66.9386
3.0	66.5224	62.8365	61.9311
3.5	61.2537	58.3186	57.2124
4.0	56.8442	54.2691	53.1762
4.5	52.9198	50.6043	49.5929
5.0	49.5119	47.4286	46.5536

**Table 6:** Results using the Epoc dataset. The provided CRR (%) values are averaged for each frequency at TW = 5 s. Note: The bold value indicates the best value on each frequency.

Frequency Stimulus (Hz)	DFN	SVM-FBCSP	kNN-FBCSP
12	**95.9091**	94.5455	89.5455
10	**90.4545**	89.5455	81.3636
8.57	**94.0909**	89.5455	85.4545
7.50	**91.8182**	87.7273	81.8182
6.66	**94.5455**	90.9091	83.1818
average	**93.3636**	90.4545	84.2727

**Table 7:** Results using the Epoc dataset. The provided CRR (%) values are averaged over all frequencies for various TW. Note: The bold value indicates the best value on each TW.

TW (s)	DFN	SVM-FBCSP	kNN-FBCSP
0.5	**72.1818**	72.0909	70.0000
1.0	82.0000	**83.0000**	77.0909
1.5	**88.1818**	85.2727	79.0000
2.0	**89.4545**	86.8182	81.1818
2.5	**91.2727**	87.7273	82.4545
3.0	**90.8182**	89.1818	83.7273
3.5	**91.9091**	89.4545	83.8182
4.0	**92.3636**	90.6364	85.1818
4.5	**93.0000**	90.1818	85.0000
5.0	**93.3636**	90.4545	84.2727

**Table 8:** Results using the Epoc dataset. The utility (U) values are averaged over all frequencies for various TW. Note: The bold value indicates the best value among all TWs.

TW (s)	DFN	SVM-FBCSP	kNN-FBCSP
0.5	**24.8125**	24.7812	24.0625
1.0	23.1282	23.4103	21.7436
1.5	21.0870	20.3913	18.8913
2.0	18.5660	18.0189	16.8491
2.5	16.7333	16.0833	15.1167
3.0	14.9105	14.6418	13.7463
3.5	13.6622	13.2973	12.4595
4.0	12.5432	12.3086	11.5679
4.5	11.6250	11.2727	10.6250
5.0	10.8105	10.4737	9.7579

**Table 9:** Ablation Study using the Speller dataset. Note: The bold value indicates the best value on each TW.

TW (s)	Layers	CRR
0.5	13 (B1 + B2 + B3)	**85.3214**
0.5	10 (B2 + B3)	81.4643
0.5	7 (B3)	80.8214
1.0	13 (B1 + B2 + B3)	**95.1429**
1.0	10 (B2 + B3)	93.3452
1.0	7 (B3)	93.3810

## Data Availability

The datasets that have been used in this study are available on the Internet. The *Speller* dataset can be found at http://bci.med.tsinghua.edu.cn/download.html (accessed on 9 June 2017). The *EPOC* dataset can be found at https://physionet.org/content/mssvepdb/1.0.0/ (accessed on 3 September 2016).
